# Psychometric properties of the Spanish version of the climate anxiety scale in Spanish-speaking adolescents

**DOI:** 10.3389/fpsyg.2025.1631481

**Published:** 2025-08-05

**Authors:** David Jimenez-Vazquez, Jose-Antonio Piqueras, Lourdes Espinosa-Fernandez, Josefa Canals-Sans, Luis-Joaquin Garcia-Lopez

**Affiliations:** ^1^Division of Clinical Psychology, Department of Psychology, Universidad de Jaén, Jaén, Spain; ^2^Department of Health Psychology, Miguel Hernandez University, Elche, Spain; ^3^Department of Psychology, Universitat Rovira I Virgili, Tarragona, Spain

**Keywords:** climate anxiety, climate change, adolescents, assessment, validation

## Abstract

**Introduction:**

Evidence suggests that climate change affects both the physical and mental health of the global population. In this context, interest in research and in the development of reliable and valid tools to measure climate anxiety—defined as the experience of intense anxiety associated with perceptions of climate change—has increased.

**Methods:**

The sample consisted of 1,065 respondents (49% self-identified as females) aged between 12 and 18 years (*M* = 14.0, SD = 1.49). This study is the first to evaluate the psychometric properties of the Climate Anxiety Scale, a 13-item questionnaire designed to assess anxiety as a psychological response to climate change, in a large population of Spanish-speaking adolescents.

**Results:**

The results showed a satisfactory model fit for the scale, with two subscales (cognitive-emotional impairment and functional impairment), both demonstrating adequate internal consistency. The subscales were invariant across gender, age, and socioeconomic status. Both subscales showed weak positive correlations with measures of emotional symptoms, emotional dysregulation, quality of life, and resilience—particularly for the functional impairment subscale. Network analyses indicated low centrality and connectivity of the CAS total score and subscales within the system. Overall levels of climate anxiety were low, though higher levels were observed among female adolescents, younger adolescents, and those from families with lower socioeconomic status.

**Discussion:**

This study provides support for the use of the CAS in the Spanish-speaking adolescent population. The findings suggest that adolescent climate anxiety functions independently and reflects a complex emotional and existential response to the ecological crisis.

## 1 Introduction

There is clear evidence that climate change has direct and indirect effects on the health of populations across the world, being a great challenge of the 21st century (Villalobos-Prats et al., 2023). Consequently, addressing its impacts requires a multidisciplinary approach, where environmental psychology and the study of pro-environmental behaviors play a key role (López-Cabanas et al., 2019). The impact on human health encompasses not only physical health (Costello et al., 2023) but also mental health (Hogg et al., 2024; Hwong et al., 2022; Nori-Sarma and Galea, 2024). Results from the United Nations Intergovernmental Panel on Climate Change, indicate that climate change is a serious threat to children and adolescent mental health (Clemens et al., 2020). In this last field, both the awareness of the consequences related to climate change and the direct effect of climate events (e.g., subtle temperature changes) can lead to emotional responses, such as anxiety, negative mood and irritability (Hwong et al., 2022; Ramadan et al., 2023). Scientific data on the associations between climate change and mental health is growing, focusing mainly on anxiety (Heeren and Asmundson, 2023). Climate anxiety has been referred among people worldwide (Clayton et al., 2023; Ogunbode et al., 2022). Although the concept of negative emotion related to environmental loss began to be talked about almost two decades ago (Kevorkian, 2004), the terms of eco-anxiety and climate anxiety have been introduced more recently. Clayton defined eco-anxiety as a chronic fear of environmental doom (Clayton et al., 2017) and climate anxiety as the experience of intense anxiety associated with perceptions about climate change, regardless of personal experiences (Clayton, 2020). The level of severity of the climate anxiety can be related to individual and sociodemographic risk factors but also is related to positive or negative psychological effects. Data gathered in 32 countries showed climate anxiety was negatively associated with mental wellbeing (Gago et al., 2024; Ogunbode et al., 2022).

In recent years, there has been a growing interest in the young population. Data have revealed that children and adolescents may be more likely to fear and worry about climate change than other age groups affecting their health and well-being (Proulx et al., 2024). Youth has also been found to be a particularly vulnerable group as are those with the highest rate of negative emotional responses associated with climate change (Boluda-Verdú et al., 2022; Cianconi et al., 2023; Clayton et al., 2023; Patrick et al., 2022; Proulx et al., 2024). For instance, young Canadians have reported that climate change affects their mental health by 78% (Galway and Field, 2023) and participants from another 10 countries reported to be very or extremely worried and were at least moderately worried about climate change (Australia, Brazil, Finland, France, India, Nigeria, Philippines, Portugal, the United Kingdom, and the United States) (Hickman et al., 2021). Recently, Teo et al. (2023) reported that one in four Australians aged 15 to 19 felt very or extremely concerned about climate change. Climate anxiety has also found to contribute to young people having negative perspectives for the future (Boluda-Verdú et al., 2022; Hickman et al., 2021; Teo et al., 2023) and increases the risk of emotional problems (Ramadan et al., 2023). Using longitudinal studies, literature have found that adolescents aged 10–11 who had high levels of worry about climate change over time presented with high rates of depressive symptoms when assessed at their late adolescence (Sciberras and Fernando, 2021). Understanding adolescents’ concerns about climate change is crucial, as they can become active social agents in mitigating the problem by developing competencies that promote action both in the present and in the future, while also learning psychological coping strategies that help them face perceived climate change threats and, consequently, reduce the risk of developing psychopathology (Daeninck et al., 2023; Ojala, 2022).

However, there is little evidence on the relationship between climate-related negative emotions and mental health in adolescence in Spanish-speaking adolescents as a result of lacking well-validated climate anxiety measures. For the first time, this paper is aimed at examining the psychometric properties of the Climate Change Anxiety Scale (CAS) developed by Clayton and Karazsia in 2020 in a large population of Spanish-speaking adolescents. The CAS is a 13-items scale, with two subscales named as Cognitive-emotional impairment and Functional impairment. In adults, data have partially supported the initial factorial structure. On the one hand, some authors have replicated the two-factor structure in France (Mouguiama-Daouda et al., 2022), Korea (Jang et al., 2023), Slovenia (Plohl et al., 2023), Arab countries (Fekih-Romdhane et al., 2024) and also in the multi-country study performed by Tam et al. (2023) in India, China, Japan and USA. These last authors demonstrated that this measure exhibited configural and metric invariance in the four countries and they suggested that the CAS can be used to assess climate change anxiety across countries. However, other studies have found different factorial solutions in samples from various countries. In Australia, Hogg et al. (2023) concluded that while the CAS is generally a valid measure, its factorial structure shows some ambiguity. In Italy, Innocenti et al. (2021) supported the two-factor model but hypothesized a possible unifactorial structure—an approach that was also observed in the Polish study, where a single-factor solution was recommended (Larionow et al., 2022). Similarly, the German study (Wullenkord et al., 2021) failed to replicate the original structure of cognitive-emotional and functional impairment. In the United States, Cruz and High (2022) also noted some ambiguity in the data, suggesting that a unifactorial structure might be more appropriate, although they still regarded the CAS as a generally valid instrument. In other cultural contexts, model modifications have been proposed to improve fit. For instance, in Indonesia, Jaro’ah and Saffana (2023) retained the two-factor structure after implementing adjustments that led to a reasonable model fit. Likewise, in the Philippines, Simon et al. (2022) used the two-factor structure but introduced correlations between four error terms to improve model fit.

Evidence regarding the measurement invariance of the scale across demographic groups has been mixed. Fekih-Romdhane et al. (2024) demonstrated configural, metric, and scalar invariance across gender, suggesting that the scale functions equivalently for males and females. In contrast, Hogg et al. (2023) found configural invariance across gender but did not confirm metric or scalar invariance, indicating potential differences in how the construct is interpreted or responded to by different gender groups. However, Larionow et al. (2022) reported full measurement invariance—configural, metric, and scalar—across gender, age, and educational level, supporting the generalizability of the scale across these demographic categories. Given these inconsistencies, further research is recommended to expand the evidence on measurement invariance across diverse population groups.

With the above-mentioned data, this study aimed to examine the psychometric properties of the Climate Anxiety Scale (CAS) in Spanish-speaking adolescents presenting evidence on the factorial structure, reliability and validity. Furthermore, connections of the climate anxiety with psychological variables were explored using network analysis. Due to the limited data of climate anxiety in Spanish-speaking adolescents, validating this well-known tool to assess climate change anxiety could assist researchers and clinicians to collect data and provide to adolescents with coping strategies to enhance emotional well-being and prevent emotional problems, particularly in those at risk.

## 2 Method

### 2.1 Participants

The sample was composed by 1,065 Spanish-speaking adolescents (49% self-identified as girls and 51% as boys) ranging from 12 to 18 years old (*M* = 14.0; *DT* = 1.49). Nine hundred and twenty participants (88.4%) had Spanish citizenship and 11.6% were migrant from Latin America (5.4%), Western Europe (2.7%), Eastern Europe (1.9%), Asia (1.1%), Africa (0.3%) and others regions (0.2%). The ethnic composition of the sample was similar to data from the general population in Spain (13.4%; Instituto Nacional de Estadística (INE), 2024).

### 2.2 Measures

The Climate Anxiety Scale (CAS; Clayton and Karazsia, 2020) is a 13-item questionnaire assessing anxiety related to climate change, divided into two subscales: cognitive-emotional impairment (8 items, e.g., “Thinking about climate change makes it difficult for me to sleep”) and functional impairment (5 items, e.g., “I have problems balancing my concerns about sustainability with my family’s needs”). Respondents rate statements on a 5-point Likert scale (1 = never, 5 = almost always). Subscale scores can be used independently or combined for a global score.

The Strengths and Difficulties Questionnaire^1^ (SDQ, Goodman, 1997), both the parent and self-version of the Emotional Subscale, using a 3-point Likert scale (0–2). Both versions show adequate psychometric properties and cut-off scores in Spanish-speaking population (Barriuso-Lapresa et al., 2014; Ortuño-Sierra et al., 2014). This study found good reliability for both self-report (*α* = 0.82, *ω* = 0.83) and parent versions (*α* = 0.80, ω = 0.80), similar to found by other studies with Spanish-speaking adolescents (Garcia-Lopez et al., 2024; Vivas-Fernandez et al., 2023a,b).

The Revised Child Anxiety and Depression Scale (RCADS-30, Sandín et al., 2010) is a 30-item questionnaire using a Likert scale from 0 to 3, assessing anxiety and depression symptoms in children and adolescents across subscales: panic disorder (PD), social phobia (SoP), separation anxiety disorder (SAD), generalized anxiety disorder (GAD), obsessive-compulsive disorder (OCD), and major depressive disorder (MD). The RCADS-30 shows excellent psychometric properties (Piqueras et al., 2017). In our study, the total score demonstrated high reliability (*α* = 0.94, *ω* = 0.94), with subscale reliabilities ranging from acceptable to good (*α* = 0.64 to 0.87, *ω* = 0.67 to 0.87), comparable to findings reported in prior research with Spanish-speaking adolescents (Garcia-Lopez et al., 2024; Piqueras et al., 2017; Vivas-Fernandez et al., 2023a,b).

The 10-Item Connor-Davidson Resilience Scale (CD-RISC-10; Campbell-Sills and Stein, 2007) is a shortened self-report version of the original scale, assessing resilience through 10 items on a Likert scale from 0 to 4. The Spanish version shows good internal consistency (α = 0.85) and is a reliable, valid measure (Notario-Pacheco et al., 2011). In this study, reliability was also good (*α* = 0.87, *ω* = 0.87), consistent with previous data from Spanish-speaking adolescents (Garcia-Lopez et al., 2024; Vivas-Fernandez et al., 2023a,b).

The KIDSCREEN-10 Index (Ravens-Sieberer et al., 2001) assesses the overall health-related quality of life of children and adolescents in terms of physical, mental, and social health. It consists of 10 items with a Likert-type response format ranging from 0 to 5. The KIDSCRREN-10 has shown good psychometric properties across European countries and for Spanish-speaking adolescents in particular (Ravens-Sieberer et al., 2010). In this study, the Cronbach’s alpha value was 0.83 and the McDonald’s omega was 0.84. These results align with those observed in earlier studies involving Spanish-speaking adolescents (Garcia-Lopez et al., 2024; Vivas-Fernandez et al., 2023a,b).

The Difficulties in Emotion Regulation Scale (DERS; Gratz and Roemer, 2024). It assesses emotional regulation through 36 items with a Likert-type response format ranging from 0 to 4 grouped into six dimensions (non-acceptance of emotional responses, difficulties in directing behavior toward goals when upset, difficulties in controlling impulsive behaviors when upset, effective emotional regulation strategies, lack of emotional awareness and emotional clarity). The Spanish adaptation was applied which has shown adequate psychometric properties in Spanish adolescents (Hervás and Jodar, 2008). Good internal consistency values were found in this study: 0.87 and 0.88 for Cronbach’s alpha and the McDonald omega, respectively.

In addition, an ad-hoc questionnaire was used to assess the sociodemographic variables of the sample, including age, gender, and the subjective socioeconomic level of the household (stratified as low, middle, or high).

### 2.3 Translation procedure

Two bilingual researchers in Spanish and English independently translated the original items of the CAS from English to Spanish, resolving any disagreements. The Spanish items were then back-translated into English by other researchers who were unaware of the original scale. Finally, the wording of the Spanish items was refined. The translation of the scale aimed for simple syntax and avoided the use of complex terms.

### 2.4 Research procedure

Upon approval from the Ethics Committee of the University of Jaén (ID: GEN-3461-aab8-41a3-85c2-ca28-5102-cdda-8d53), the assessment protocol was administered online in 2023 using the Google Forms platform. The dissemination and recruitment for this study were primarily conducted through secondary education centers in Spain, but also through social media and other forms of communication. Informed consent was obtained from the legal guardians and the adolescents themselves in an online format through a secure platform. Participants received no compensation for filling out the assessment battery.

### 2.5 Data analysis

The analyses for calculating descriptive statistics, reliability analysis of the scales using Cronbach’s Alpha and McDonald’s Omega, and confirmatory factor analyses (CFA) were conducted using the JASP 0.18.1 program (JASP Team, 2023). For the CFA, the following fit indices were considered: root mean square error of approximation (RMSEA), standardized root mean square residual (SRMR), comparative fit index (CFI), Tucker–Lewis index (TLI), and Akaike information criterion (AIC). RMSEA and SRMR values ≤ 0.08 indicate acceptable fit. For the CFI and TLI indices, values ≥ 0.9 are considered acceptable (Hu and Bentler, 1999). The CAS factor models were compared using the AIC, where lower AIC values indicate better fit (Byrne, 2013).

Similar to the procedure followed in previous studies that validated the CAS in other population samples and examined the invariance of the scale scores (Fekih-Romdhane et al., 2024; Hogg et al., 2023; Larionow et al., 2022), the present study investigates measurement invariance in an adolescent population, considering gender (self-identified boys and girls), age group (12–14 and 15–18 years), and socioeconomic status (low, medium, and high). Measurement invariance was assessed using the established multi-step procedure proposed by Van de Schoot et al. (2012), which involves comparing nested models with progressively constrained parameters and analyzing changes in model fit. To this end, we conducted a multi-group CFA, evaluating measurement equivalence at the configural (same factorial structure), metric (equal factor loadings), and scalar (equal item intercepts) levels (Vandenberg and Lance, 2000). Invariance was considered to be present when the change in CFI was ≤0.01 and the change in RMSEA was ≤0.015 (Chen, 2007). Comparisons between sociodemographic variables were calculated using the Mann–Whitney *U* test and the Kruskal–Wallis test (for non-parametric comparisons due to violations of normality assumptions). *Post hoc* analysis was performed using the Mann–Whitney *U* test.

Network analysis was also employed as a complementary approach to traditional correlational methods, with the aim of visually and structurally exploring the relationships between climate anxiety and various psychological variables in adolescence, using JASP 0.18.1 software (JASP Team, 2023). The use of network analysis has grown exponentially in the study of emotional psychopathology, driven by increasing support for the conceptualization of emotional difficulties as functionally interconnected elements (Cai et al., 2020; McNally, 2021). Network analysis allows variables to be represented as nodes and their associations as edges, facilitating the identification of the most central and influential variables within the network and enhancing the understanding of complex associations among psychological constructs (Borsboom and Cramer, 2013; Epskamp et al., 2018). Among centrality indices, *betweenness* measures how often a node lies on the shortest paths between other nodes, highlighting variables that function as critical bridges or connectors. *Closeness* reflects how close a node is to all other nodes, indicating variables that can quickly influence or be influenced by the rest of the network. *Strength* quantifies the sum of the weights of the edges connected to a node, capturing the overall direct influence or connectivity of that variable.

Given that emotion regulation has gained increasing importance in research on the psychological consequences of climate change perceptions—as demonstrated by recent studies (Ejelöv et al., 2018; Edizioni Centro Studi Erickson, 2022; Ojala, 2012)—and that symptoms of anxiety and depression have been consistently associated with concerns about its consequences (Hickman et al., 2021), the present study includes, for the network analyses, measures of emotional symptoms assessed through the RCADS-30 subscales and emotion regulation difficulties evaluated with the DERS.

## 3 Results

### 3.1 Structural validity and reliability

The Shapiro–Wilk multivariate normality test indicated the absence of normality of the CAS items. Due to this, robust maximum likehood (robust ML) estimation was conducted. The description of models and the goodness-of-fit indices can be found in Table 1. Factor loadings (all *p*s < 0.001) are presented in Table 2. Confirmatory Factor Analysis revealed that the original bifactorial model was replicated in this study, with acceptable indices except for RMSEA. The estimated covariance between subscales was high (0.85; *p* < 0.001). The goodness-of-fit improved after the correlation between the measurement errors of items 1 and 2 was specified.

**Table 1:** Goodness-of-fit indices for the CAS models (robust ML estimation).

Models	χ^2^*/df*	CFI	TLI	RMSEA (90% CI)	SRMR	AIC
2-factor model	726.274/64 = 11.34	0.88	0.86	0.010 (0.092; 0.105)	0.055	20566.812
2-factor model (correlating measurement errors 1–2)	432.939/63 = 6.87	0.94	0.92	0.074 (0.068–0.081)	0.043	20141.267

Factor 1: Cognitive-emotional impairment (items 1–8) factor 2: functional impairment (items 9–13).

**Table 2:** Descriptive statistics of the Climate Anxiety Scale (CAS) statements and standardized factor loadings from the CFA.

Item	*M*	*SD*	Skewness	Kurtosis	ITC	2-factor model	2-factor model (item 1–2)
1	1.51	0.908	1.932	3.402	0.71	0.61	0.55
2	1.40	0.798	2.274	5.238	0.70	0.68	0.64
3	1.20	0.550	3.300	12.785	0.51	0.64	0.64
4	1.17	0.559	3.823	16.712	0.50	0.68	0.69
5	1.50	0.926	1.856	2.761	0.66	0.59	0.58
6	1.14	0.486	3.892	17.663	0.50	0.73	0.73
7	1.13	0.519	4.560	23.442	0.42	0.57	0.59
8	1.23	0.632	3.156	11.006	0.56	0.66	0.67
9	1.17	0.545	3.795	16.966	0.50	0.70	0.70
10	1.39	0.784	2.187	4.703	0.58	0.53	0.53
11	1.13	0.503	4.754	25.840	0.46	0.78	0.78
12	1.11	0.460	4.997	29.577	0.42	0.77	0.77
13	1.10	0.474	5.336	32.289	0.40	0.66	0.65

Robust maximum likelihood estimation method (ML), all *p* < 0.001; ITC, item-total correlation.

Internal consistencies for the CAS in our sample were very good. For overall score of the CAS: *α* = 0.88 and *ω* =0.88 were found. For the cognitive-emotional impairment subscale, data revealed levels of internal consistency as follows: *α* = 0.84 and *ω* = 0.85, whereas it was found that functional impairment subscale presented with *α* = 0.80 and *ω* = 0.80.The mean score for each item was 1.10–1.51. The skewness and the kurtosis values ranged from 1.86 to 5.33 and 2.76 to 23.44, respectively. The item-total correlation coefficient revealed that the correlation coefficient of all the items was 0.40–0.71.

### 3.2 Measurement invariance

A series of invariance analyses were conducted to assess the configural, metric and scalar invariance of CAS across gender [self-identified as female (*N* = 544) vs. self-identified as male (*N* = 520)], age [12–14 (*N* = 710) vs. 15–18 (*N* = 355)] and socioeconomic status [low (*N* = 166), medium (*N* = 718), high (*N* = 181)]. Overall, the 2-factor correlated model was invariant for age, gender and socioeconomic status (please, see Table 3).

**Table 3:** Goodness-of-fit indices for invariance analysis of the CAS in terms of socio-demographic variables. 2-factor correlated model (correlating measurement errors item 1–2).

	χ^2^(df)	CFI	ΔCFI	RMSEA	ΔRMSEA
Variable 1: Gender (females and males)					
Configural invariance	641.456 (126)	0.907	–	0.088	–
Metric invariance	760.363 (137)	0.897	−0.010	0.092	0.004
Scalar invariance	799.254 (148)	0.892	−0.005	0.091	−0.001
Variable 2: age (12–14 and 15–18 years old)					
Configural invariance	689.159 (126)	0.907	–	0.092	–
Metric invariance	706.611 (137)	0.906	−0.001	0.088	−0.004
Scalar invariance	717.807 (148)	0.906	0	0.085	−0.003
Variable 3: socioeconomic status (low, medium, high)					
Configural invariance	903.495 (189)	0.884	–	0.103	–
Metric invariance	982.097 (211)	0.875	−0.009	0.101	−0.002
Scalar invariance	1010.304 (233)	0.874	−0.001	0.097	−0.004

### 3.3 Convergent and divergent validity

Functional impairment subscale showed significant positive correlations, with medium effect sizes, with the self-reported SDQ-Emotional subscale, the DERS, as well with both the RCADS Total score and the panic, social phobia and obsessive-compulsive disorder subscales. Statistically significant positive correlations were found between the scores of the cognitive-emotional impairment subscale and emotional risk measures (SDQ) and the RCADS subscales. Statistically significant negative correlations between the cognitive-emotional impairment subscale and resilience and quality of life were also found. Similarly, significant correlations were found with the functional impairment subscale measure, with moderately higher effect sizes compared to those observed with the previous subscale (please, see Table 4).

**Table 4:** Spearman’s correlations between the original CAS subscales and the analyzed variables.

Scales	Overall CAS score	Cognitive-emotional impairment subscale (factor 1; 1–8 items)	Functional impairment subscale (factor 2; 9–13 items)
Self-Report emotional SDQ	0.27***	0.20***	0.31***
Parent emotional SDQ	0.10***	0.07*	0.13***
RCADS-30 (total)	0.34***	0.26***	0.36***
RCADS (MD)	0.23***	0.14***	0.28***
RCADS (PD)	0.27***	0.20***	0.32***
RCADS (SoP)	0.29***	0.23***	0.30***
RCADS (SAD)	0.24***	0.23***	0.18***
RCADS (GAD)	0.30***	0.26***	0.27***
RCADS (OCD)	0.29***	0.21***	0.32***
CD-RISC-10	−0.14***	−0.09**	−0.17***
KIDSCREEN-10	−0.18***	−0.11***	−0.26***
DERS	0.22***	0.15***	0.31***

*p ≤ 0.05, **p ≤ 0.01, ***p ≤ 0.001.

### 3.4 Climate anxiety in Spanish adolescent population

Table 5 presents the descriptive statistics and differences based on gender, age ranges (evaluated using the Mann–Whitney *U* test), and subjective socioeconomic status (evaluated using the Kruskal-Wallis H test) for all the variables included in this study. Female adolescents scored significantly higher than their male peers in cognitive impairment, functional impairment, and the global CAS score. Regarding age, significant differences were observed as younger adolescents (12–14 years) scored significantly higher in both the cognitive impairment subscale and the global CAS score, with no significant differences in the functional impairment subscale. Differences were found in functional impairment and overall climate anxiety scale on the SES. Thus, adolescents with lower SES showed significantly higher levels of functional impairment compared to those with medium or high SES, with small effect sizes. Adolescents with lower SES reported higher climate anxiety than those with high SES, with small effect sizes.

**Table 5:** Descriptive statistics and group differences by sex, age, and subjective socioeconomic status.

	Total sample (*N* = 1,065)	Gender			Age			Socioeconomic status (SES)				
		Females (*n* = 520)	Males (*n* = 545)	*p*-value (Mann–Whitney *U*-test)	12–14 years old (*n* = 710)	15–18 years old (*n* = 355)	*p*-value (Mann–Whitney *U*-test)	Low (*n* = 166)	Medium (*n* = 718)	High (*n* = 181)	*p*-value (Kruskal–Wallis*H*-test)	*Post hoc*: *p*-value (Mann–Whitney *U*-test)
	*M* (SD)	*M* (SD)			*M* (SD)			*M* (SD)				
Cognitive-emotional impairment	10.29 (3.80)	10.50 (3.97)	10.09 (3.63)	.040^a1^	10.45 (3.82)	9.97 (3.76)	0.007 ^a1^	10.53 (3.91)	10.31 (7.72)	9.98 (4.02)	*ns*	N/A
Functional impairment	5.90 (2.08)	6.05 (2.11)	5.76 (2.05)	<0.001 ^a1^	5.95 (2.13)	5.79 (1.98)	*ns*	6.39 (2.67)	5.82 (1.89)	5.76 (2.15)	.001^b1^	Low > Medium: 0.001 ^a1^ Low > High: 0.001 ^a1^ Medium – High: *ns*
Overall score of the CAS	16.19 (5.44)	16.55 (5.59)	15.84 (5.27)	0.003 ^a1^	16.40 (5.50)	15.76 (5.29)	0.014 ^a1^	16.92 (6.16)	16.13 (5.15)	15.74 (5.78)	0.026 ^b1^	Low – Medium: *ns* Low > High: 0.007 ^a1^ Medium – High: *ns*

ns: not significant; Effect size: Pearson’ *r* (non-parametric Mann–Whitney *U*-Test): small (a1) = 0.1, medium (a2) = 0.3, large (a3) = 0.5; η^2^ (non-parametric Kruskal–Wallis *H*-test): small (b1) = 0.01, medium (b2) = 0.0, large (b3) = 0.14.

**Figure 1:**
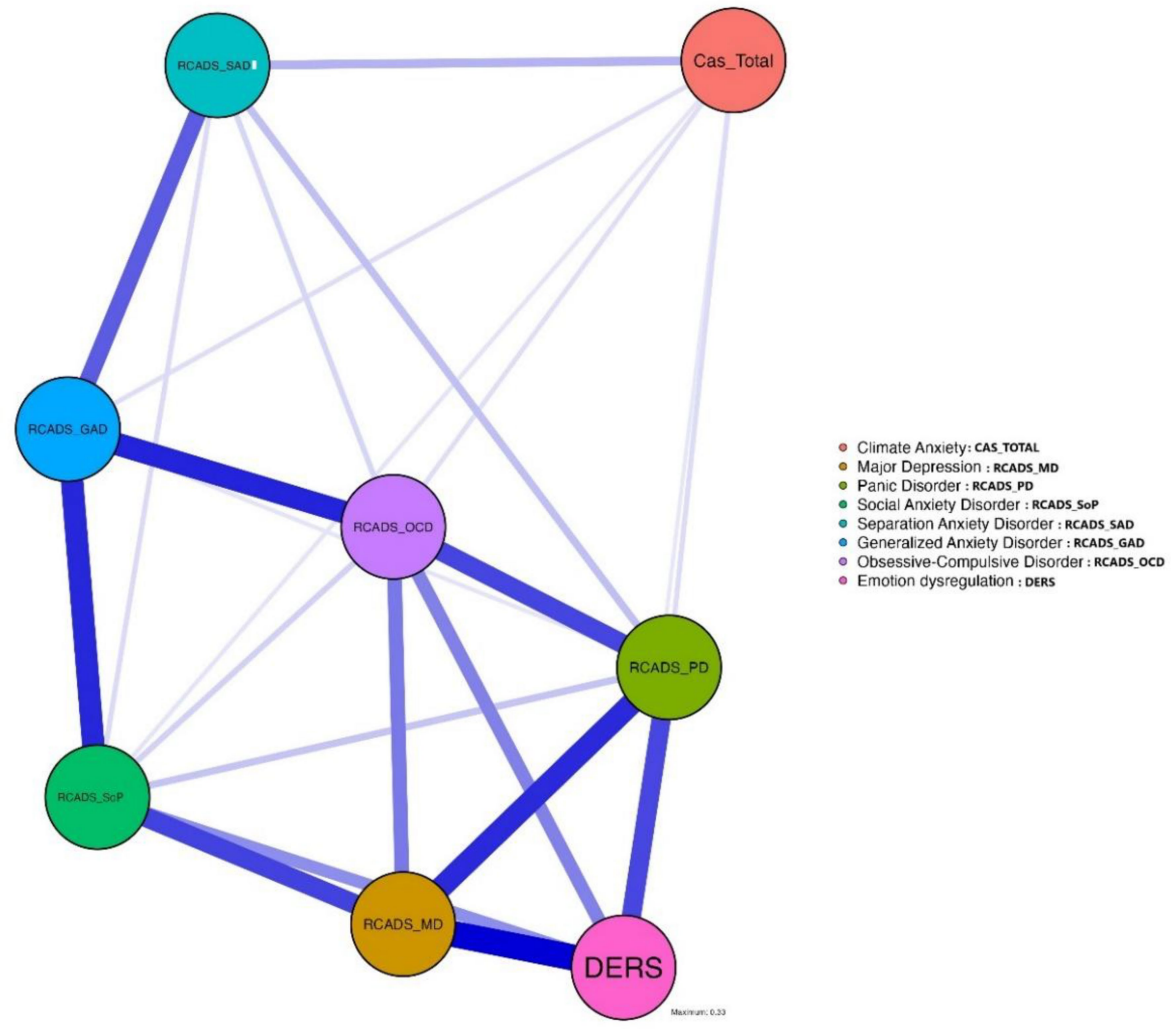
Network analysis of relationships between variables and overall CAS score.

The network analysis explored the relationship between the CAS (overall score and subscales) and both emotional symptoms (measured by RCADS subscales) and emotion dysregulation (measured by DERS). First, as far as CAS overall score is concerned, the network structure consisted of 8 nodes and 24 significant edges out of a possible 28, resulting in a sparsity of 0.143, indicating a relatively dense network. Regarding the centrality of CAS Total Score, it occupied a peripheral position in the network: Betweenness: −0.846, reflecting a limited role as a mediator; Closeness: −2.153, indicating low integration within the network; and Strength and Expected Influence: both at −1.916, suggesting weak connectivity and influence. The key nodes in the network were Generalized Anxiety, which emerged as the most central variable with the highest betweenness (1.974) and closeness (0.980), acting as a hub connecting other symptoms. Similarly, Major Depression and Obsessive-Compulsive symptoms demonstrated high strength (0.819 and 0.766, respectively), underlining their importance in the emotional network. The CAS Total Score exhibited weak connections with other variables, most notably with Panic Symptoms (*r* = 0.045). It showed minimal links with Generalized Anxiety (*r* = 0.041) and Emotion Dysregulation (*r* = 0.029) (see Figure 1). Regarding Emotion Dysregulation, it displayed moderate strength (0.378) and some relevance within the network. However, its direct connection with the CAS Total Score was weak, indicating a limited impact of climate anxiety on emotion dysregulation in this context. In summary, the CAS Total Score played a marginal role within the emotional network, showing low centrality and weak connections with other symptoms, including emotion dysregulation. In contrast, generalized anxiety and major depression emerged as pivotal nodes, emphasizing their centrality in the network and their significance in understanding emotional dynamics. These findings suggest that climate anxiety operates more independently within the emotional system analyzed (see Supplementary Table 1 and Supplementary Figure 1).

**Figure 2:**
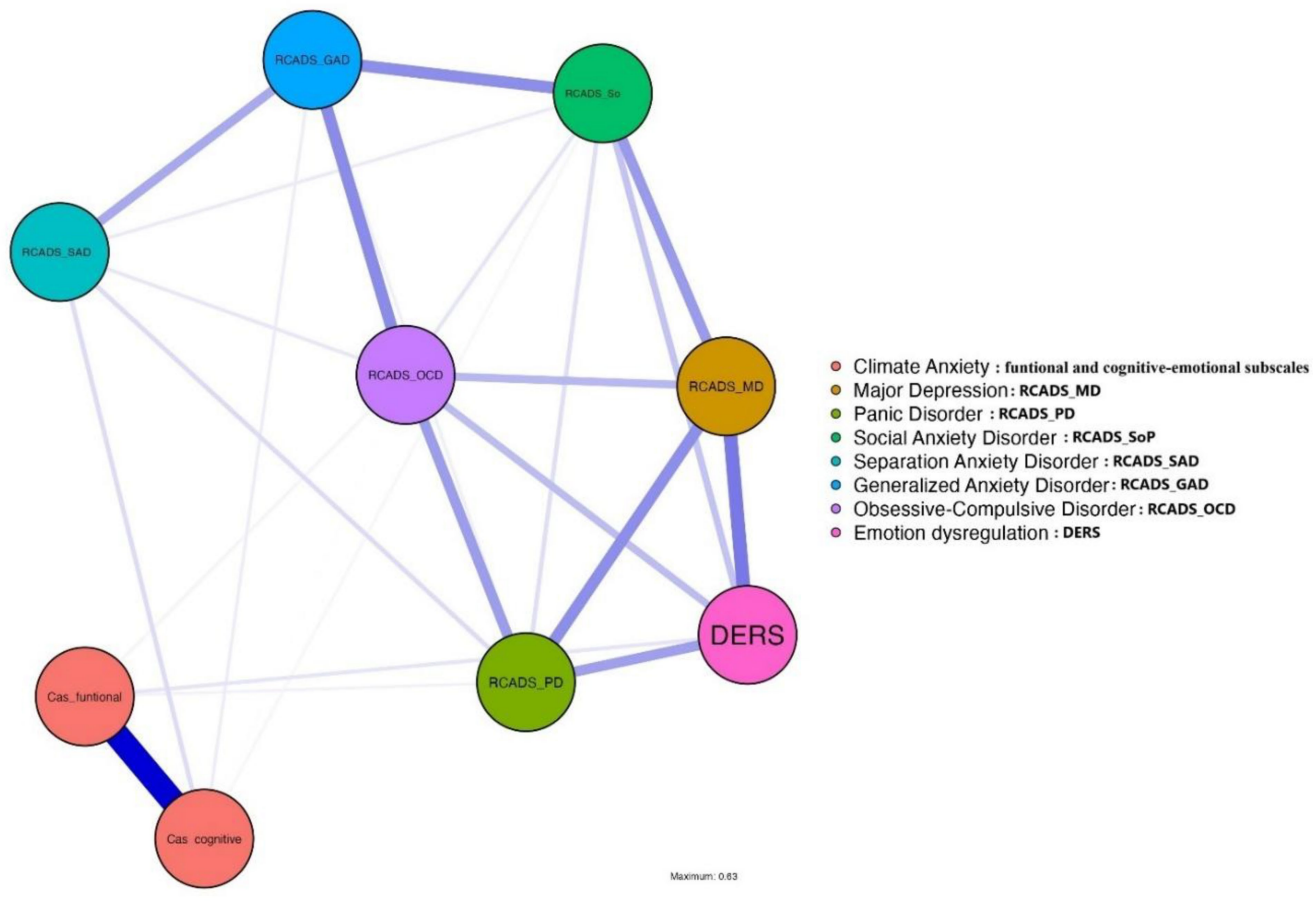
Network analysis of relationships between variables and CAS subscales: cognitive-emotional impairment and functional impairment.

Second, the associations between the cognitive-emotional and functional dimensions of CAS and emotional symptoms and emotion dysregulation were explored. The network comprised 9 nodes and 25 significant edges out of a possible 36, resulting in a sparsity of 0.306, indicating moderate density. Both cognitive and functional dimensions of CAS were peripheral in the network. CAS Cognitive exhibited low betweenness (0.165), closeness (−1.683), and strength/expected influence (−0.435), reflecting weak integration, mediation, and influence. Similarly, CAS Functional demonstrated even lower betweenness (−0.206), closeness (−1.719), and strength/expected influence (−0.623), highlighting its marginal role (see Figure 2). In contrast, Generalized Anxiety emerged as the most central node, with the highest betweenness (2.018) and closeness (1.007), serving as a critical connector. Major Depression and Obsessive-compulsive symptoms also displayed strong centrality, with high strength values (1.032 and 0.878, respectively). The CAS dimensions exhibited weak relationships with other variables: CAS Cognitive was minimally connected to Social Phobia; (*r* = 0.018) and Generalized Anxiety (*r* = 0.041), while CAS Functional had limited connections to DERS (*r* = 0.059) and Panic Symptoms; (*r* = 0.030). Emotion Dysregulation showed moderate strength (0.480) within the network but maintained limited ties with the CAS dimensions. In summary, the cognitive and functional dimensions of CAS displayed low centrality and weak connectivity within the network, suggesting they function more independently in emotional dynamics. By contrast, generalized anxiety and major depression emerged as pivotal nodes, underscoring their significant roles in understanding emotional symptom networks (see Supplementary Table 2 and Supplementary Figure 2).

## 4 Discussion

This is the first study to examine the psychometric properties of the Climate Anxiety Scale, potential correlates and the impact of climate anxiety in Spanish-speaking adolescent population. Data supported the 2-factor model proposed by the original authors, consistent with other studies with adult population who also found difficulties replicating a 2-factor model (Fekih-Romdhane et al., 2024; Jang et al., 2023; Jaro’ah and Suffana, 2023; Mouguiama-Daouda et al., 2022; Plohl et al., 2023; Simon et al., 2022; Tam et al., 2023). The present study has also proved CAS to be configural, metric and scalar invariant across gender, age and SES. Compared with literature with adults, Fekih-Romdhane et al. (2024) have reported the configural, metric and scalar invariance of the scale across gender, whereas Larionow et al. (2022) found it not gender but age as well. However, Hogg et al. (2023) demonstrated the metric and scalar invariance across age but only configural invariance was demonstrated across gender. In a study with adult population across four countries, Tam et al. (2023) tested the measurement invariance of the scale and concluded that the configural and metric invariance models were acceptable, but the scalar invariance model was rejected. In our study, invariance across age, gender and SES implies that the same latent variable structure retains between female and male adolescents, different age period and SES. Further studies with adolescent population are encouraged to replicate data in other countries to demonstrate the configural, metric and scalar invariance of CAS across age and SES.

The results of this study show good internal consistency of the Climate Anxiety Scale (CAS) in Spanish-speaking adolescents. The total score yielded an *α* = 0.88, in line with the ranges reported in previous, ranging from *α* = 0.84 (Jang et al., 2023) to *α* = 0.96 (Fekih-Romdhane et al., 2024). The cognitive-emotional impairment subscale showed an *α* = 0.84, placing it between the values found by Innocenti et al. (2021; *α* = 0.78) and Fekih-Romdhane et al. (2024; *α* = 0.91). Meanwhile, the functional impairment subscale yielded an *α* = 0.80, also within the range observed in the literature (*α* = 0.77 in Innocenti et al., 2021; *α* = 0.93 in Fekih-Romdhane et al., 2024).

Further, CAS was positively correlated to a higher risk of developing emotional problems (either self-reported by adolescents or reported by parent), with small to medium effect sizes. Similarly, the CAS showed statistically significant positive correlations between CAS and the current presence of mood and anxiety symptomatology measured by RCADS-30, with a medium effect size. These correlations were particularly larger in the functional impairment subscale, except for the separation anxiety subscale. This may suggest that the presence of functional impairment, caused by the perception of the consequences of climate change, could be associated with increased emotional symptomatology. Depression correlated with the CAS (overall score and subscales) but only small effect size was found. These data are aligned with findings reported by authors such as Wullenkord et al. (2021) and Innocenti et al. (2021) who reported CAS overall score was related to anxiety and depressiveness and Fekih-Romdhane et al. (2024) who found higher CAS scores (particularly, in the functional impairment subscale) were significantly associated with higher depression and anxiety symptomatology, with medium effect sizes. However, Larionow et al. (2022) and Mouguiama-Daouda et al. (2022) reported CAS was positively correlated with depressive symptoms but not with anxiety. This suggests the cognitive-emotional impairment subscale may not reflect the full spectrum of cognitive aspects of climate anxiety, as suggested by some authors (Wullenkord et al., 2021), or CAS may be measuring the emotional and cognitive response (not unequivocally maladaptive) related to climate change, as proposed by Larionow et al. (2022). The significant associations between the CAS and higher levels of symptoms of depression and anxiety in adolescents do not necessarily imply that climate anxiety should be understood as a manifestation of general depression or anxiety, or vice versa. The findings suggest that climate anxiety may play a specific and distinct role in adolescents’ emotional functioning, acting as a unique psychological phenomenon that can impact well-being in ways that differ from more common emotional disorders. This distinctive nature is especially evident in the functional impairment subscale of the CAS, which assesses how concerns about climate change interfere with concrete aspects of daily life, such as social functioning. Therefore, its association with depressive or anxious symptoms may stem from this specific functional impact.

In this regard, the results of the network analysis offer valuable insights into the emotional dynamics associated with climate anxiety. The low centrality and connectivity of the CAS overall score and subscales within the system suggest that they operate relatively independently, exerting limited influence on other emotional symptoms and emotion dysregulation. This peripheral position may indicate that, although climate anxiety is a significant phenomenon, its effects on the broader emotional system are less integrative or mediatory compared to other central emotional symptoms. In contrast, the prominence of generalized anxiety and major depression as central nodes highlight their fundamental role in the emotional structure, acting as key points of connection among symptoms. These findings emphasize the need to prioritize the management of generalized anxiety and depression in addressing complex emotional networks, and they suggest that climate anxiety may require targeted interventions less dependent on general emotion regulation dynamics. A study by Clayton and Karazsia (2020) examining climate anxiety through a validated scale highlights that its cognitive and functional dimensions are distinct and not strongly correlated with general depression or anxiety but do contribute to emotional responses to climate change (Clayton and Karazsia, 2020). Similarly, research validating the CAS in a German sample found weak associations between climate anxiety and other psychological factors like depression and generalized anxiety, suggesting also that climate anxiety functions as a unique emotional construct (Wullenkord et al., 2021). These findings align with the current analysis, which places the cognitive and functional dimensions as well as the total score of CAS in a peripheral network role, showing weak connectivity with broader emotional systems and symptoms. Together, these studies reinforce the idea that climate anxiety operates as a relatively independent construct within psychological networks, distinct from generalized anxiety and depression, and encompasses a larger gamut of emotional experiences than anxiety (e.g., Contreras et al., 2024; Dodds, 2021; Kurth and Pihkala, 2022). Our findings support the notion that climate anxiety, rather than representing a conventional psychopathological symptom, may reflect a complex emotional and existential response to the ecological crisis. This aligns with Kurth and Pihkala’s (2022) conceptualization of eco-anxiety as a form of legitimate moral distress, which calls for educational and community-based approaches rather than pathologizing clinical frameworks.

Second, climate anxiety was significantly and negatively related to resilience and quality of life of adolescents, in line with Clayton and Karazsia’s (2020) original study. Additional research has also established that the relations of climate anxiety with psychological resilience were negative and weak (Plohl et al., 2023). Third, climate anxiety was positively related to difficulties in emotion regulation in adolescents, consistent with studies with children and young people (Hickman et al., 2021) and adults (Orrù et al., 2024). With this in mind, mental health researchers may develop coping strategies by building resilience strategies and emotion regulation skills (Jimenez-Vazquez et al., 2024; Vivas-Fernandez et al., 2024). Further research is warranted.

Although research suggest the presence of a climate change generation gap whereby younger people care more about climate change than older people (Milfont et al., 2021), on average, adolescents reported low climate anxiety levels in this study (*M* = 1.25; overall score was 16.19) with significant floor effects. Moreover, adolescents in general appear to experience more cognitive-emotional impairment associated with climate change than functional impairment, perhaps because, as suggested by Chan et al. (2024), cognitive-emotional impairment may occur first, followed by functional deterioration. As this is the first study with a sample exclusively of adolescents, we could not assess whether Spanish-speaking adolescents are less or more anxious about climate change than adolescents in another cultures and languages using the CAS. However, compared to studies with participants with a mean age in the young adulthood range, Plohl et al. (2023) reported that the mean of the total CAS score was 1.65 for Slovenian young adults, Larionow et al. (2022) found an overall score of 20.34 in Poland, Fekih-Romdhane et al. (2024) revealed an overall score of 25.48 in Lebanon young adults, and up to an overall score of 28.97 in Indonesian young adults (Jaro’ah and Saffana, 2023). It is uncertain how experiences with climate change in other countries may have affected climate anxiety rates. Likewise, additional studies should further investigate whether the reformulation of the items from the original scale is a crucial aspect in improving its psychometric properties for adolescents.

In addition, self-identified females reported more climate anxiety, similar to research in adults (Boluda-Verdú et al., 2022; Clayton et al., 2023; Clayton and Karazsia, 2020; Heeren et al., 2022; Hogg et al., 2023; Larionow et al., 2022; Searle and Gow, 2010; Teo et al., 2023; Wullenkord et al., 2021). Regarding the female gender, it has also been associated with other aspects related to environmental concern, such as greater emotional affinity and lower apathy (Amérigo et al., 2012; Zelezny et al., 2000), suggesting the existence of a gender-related issue. Further, younger adolescents (12–14 years old) evidenced higher climate anxiety than older adolescents between 15 and 18 years old. This finding is aligned with data collected from a large survey which included 18,800 participants aged 15–19 years in Australia (Teo et al., 2023). Finally, in our study with adolescent population, low SES seem to be related to climate anxiety, in line with Clayton et al. (2017) who suggested that lower-income communities are more likely to have outdated infrastructure, which places these communities at greater risk for the impacts of climate change. However, our data differs from Wullenkord et al. (2021) who found climate anxiety was unrelated to income. Due to scarcity of data, it is crucial further research is conducted.

### 4.1 Limitations

Finally, some limitations must be noted. First, data did not meet the assumption of normal distribution, consistent with research with adults (Jaro’ah and Suffana, 2023; Larionow et al., 2022; Plohl et al., 2023; Wullenkord et al., 2021) but contrast with normal distribution found by Fekih-Romdhane et al. (2024). Second, this cross-sectional study has examined the psychometric properties of the CAS in a sample of Spanish-speaking adolescents. Future studies should examine the psychometric properties of CAS in adolescents from different cultures and languages, including longitudinal designs in order to characterize the developmental trajectory of climate anxiety. Emotional responses to the ecosocial crisis may be different and contingent to cultural contexts. A systematic review of this scale could rise some light as to discrepancies in the factor structure of the scale across cultures and languages. Third, the administration of the scale was online, so external factors might affect to the process of filling out the scale. Fourth, the test–retest reliability was not assessed. Finally, we cannot preclude that some differences in our findings compared to studies with adult population may be due to how youth may experience climate change distress-related impairment compared to adults. Another potential mediator to be examined is the potential impact of parent beliefs about the climate change crisis in the process of learning of his/her offspring, as Mead et al. (2012) have suggested. Future studies should address these issues.

## 5 Conclusion

There is growing evidence of the direct effects of climate change and the impact of negative emotions, such as climate anxiety, on mental health, particularly among the most vulnerable populations. In this context, interest in research and the development of reliable and valid tools to measure climate anxiety has increased. Notably, the Climate Anxiety Scale, developed less than 5 years ago, has been studied across various population groups but limited to adults. Thus, this is the first study investigating the psychometric properties of the CAS, and potential correlates of climate anxiety in a well-powered Spanish-speaking adolescent population. Our data are consistent with most studies and report that the scale is reliable and valid for use in clinical or research practice within the Spanish-speaking adolescent population. Data reveal the strong impact of climate anxiety in the emotional well-being, resilience, emotion regulation, and quality of life in adolescents. Educating the youth in their formative years on the detrimental consequences of climate change in the school curriculum to raise awareness may be beneficial. An important future direction for improving the CAS for use with adolescents is conducting qualitative research with young people from a culturally informed perspective in order to increase our understanding of adolescents’ needs and what they find most distressing about climate change.

The psychology of climate change is essential for understanding how emotions associated with this global crisis affect our mental health. Tools such as the Climate Anxiety Scale may allow researchers and clinicians for the evaluation and deeper understanding of emotional impacts, facilitating the integration of these dimensions into the design of public policies and the development of effective and sensitive interventions that address both the physical and psychological effects of climate change.

## Data Availability

The data that support the findings of this study are available from the corresponding author, upon reasonable request.

## Footnotes

^1^ www.sdqinfo.org

## References

[ref1] Amérigo M. Aragonés J. I. García J. A. (2012). Explorando las dimensiones de la preocupación ambiental. Una propuesta integradora. PsyEcology 3, 299–311. doi: 10.1174/217119712802845705

[ref2] Barriuso-Lapresa L. M. Hernando-Arizaleta L. Rajmil L. (2014). Reference values of the strengths and difficulties questionnaire (SDQ) version for parents in the Spanish population, 2006. Actas Esp. Psiquiatr. 42, 43–48. 24715361

[ref3] Boluda-Verdú I. Senent-Valero M. Casas-Escolano M. Matijasevich A. Pastor-Valero M. (2022). Fear for the future: eco-anxiety and health implications, a systematic review. J. Environ. Psychol. 84:101904. doi: 10.1016/j.jenvp.2022.101904

[ref4] Borsboom D. Cramer A. O. J. (2013). Network analysis: an integrative approach to the structure of psychopathology. Annu. Rev. Clin. Psychol. 9, 91–121. doi: 10.1146/annurev-clinpsy-050212-185608 23537483

[ref5] Byrne B. M. (2013). Structural equation modeling with AMOS: Basic concepts, applications, and programming. 2nd Edn. New York, NY: Routledge.

[ref6] Cai Y. Dong S. Yuan H. C. (2020). Network analysis and its applications in psychology. Adv. Psychol. Sci. 28, 178–190. doi: 10.3724/SP.J.1042.2020.00178

[ref7] Campbell-Sills L. Stein M. B. (2007). Psychometric analysis and refinement of the Connor-Davidson resilience scale (CD-RISC): validation of a 10-item measure of resilience. J. Trauma Stres. 20, 1019–1028. doi: 10.1002/jts.20271, PMID: 18157881

[ref8] Chan H.-W. Lin L. Tam K.-P. Hong Y.-Y. (2024). From negative feelings to impairments: a longitudinal study on the development of climate change anxiety. J. Anxiety Disord. 107:102917. doi: 10.1016/j.janxdis.2024.102917, PMID: 39217778

[ref9] Chen F. F. (2007). Sensitivity of goodness of fit indexes to lack of measurement invariance. Struct. Equ. Modeling 14, 464–504. doi: 10.1080/10705510701301834

[ref10] Cianconi P. Hanife B. Hirsch D. Janiri L. (2023). Is climate change affecting mental health of urban populations? Curr. Opin. Psychiatry 36, 213–218. doi: 10.1097/yco.0000000000000859, PMID: 36762647

[ref11] Clayton S. (2020). Climate anxiety: psychological responses to climate change. J. Anxiety Disord. 74:102263. doi: 10.1016/j.janxdis.2020.102263, PMID: 32623280

[ref12] Clayton S. Karazsia B. T. (2020). Development and validation of a measure of climate change anxiety. J. Environ. Psychol. 69:101434. doi: 10.1016/j.jenvp.2020.101434

[ref13] Clayton S. Manning C. Krygsman K. Speiser M. (2017). Mental health and our changing climate: Impacts, implications, and guidance. Washington, DC: American Psychological Association and ecoAmerica.

[ref14] Clayton S. Pihkala P. Wray B. Marks E. (2023). Psychological and emotional responses to climate change among young people worldwide: differences associated with gender, age, and country. Sustain. For. 15:3540. doi: 10.3390/su15043540

[ref15] Clemens V. Von Hirschhausen E. Fegert J. M. (2020). Report of the intergovernmental panel on climate change: implications for the mental health policy of children and adolescents in Europe—a scoping review. Eur. Child Adolesc. Psychiatry 31, 701–713. doi: 10.1007/s00787-020-01615-3, PMID: 32845381 PMC9142437

[ref16] Contreras A. Blanchard M. A. Mouguiama-Daouda C. Heeren A. (2024). When eco-anger (but not eco-anxiety nor eco-sadness) makes you change! A temporal network approach to the emotional experience of climate change. J. Anxiety Disord. 102:102822. doi: 10.1016/j.janxdis.2023.102822, PMID: 38159371

[ref18] Costello A. Romanello M. Hartinger S. Gordon-Strachan G. Huq S. Gong P.. (2023). Climate change threatens our health and survival within decades. Lancet 401, 85–87. doi: 10.1016/S0140-6736(22)02353-4, PMID: 36400092

[ref19] Cruz S. M. High A. C. (2022). Psychometric properties of the climate change anxiety scale. J. Environ. Psychol. 84:101905. doi: 10.1016/j.jenvp.2022.101905

[ref20] Daeninck C. Kioupi V. Vercammen A. (2023). Climate anxiety, coping strategies and planning for the future in environmental degree students in the UK. Front. Psychol. 14:1126031. doi: 10.3389/fpsyg.2023.1126031, PMID: 37564302 PMC10409990

[ref9001] Dodds J. (2021). The psychology of climate anxiety. BJPsych Bulletin. 45, 222–226. doi: 10.1192/bjb.2021.18 34006345 PMC8499625

[ref21] Ejelöv E. Hansla A. Bergquist M. Nilsson A. (2018). Regulating emotional responses to climate change—a construal level perspective. Front. Psychol. 9:629. doi: 10.3389/fpsyg.2018.00629, PMID: 29780340 PMC5946018

[ref22] Epskamp S. Borsboom D. Fried E. I. (2018). Estimating psychological networks and their accuracy: a tutorial paper. Behav. Res. Methods 50, 195–212. doi: 10.3758/s13428-017-0862-1, PMID: 28342071 PMC5809547

[ref23] Fekih-Romdhane F. Malaeb D. Yakın E. Sakr F. Dabbous M. Khatib S. E.. (2024). Translation and validation to the Arabic language version of the climate change anxiety scale (CCAS). BMC Psychiatry 24:507. doi: 10.1186/s12888-024-05956-0, PMID: 39014380 PMC11253455

[ref24] Gago T. Sargisson R. J. Milfont T. L. (2024). A meta-analysis on the relationship between climate anxiety and wellbeing. J. Environ. Psychol. 94, 1–14. doi: 10.1016/j.jenvp.2024.102230

[ref25] Galway L. P. Field E. (2023). Climate emotions and anxiety among young people in Canada: a national survey and call to action. J. Clim. Change Health 9:100204. doi: 10.1016/j.joclim.2023.100204, PMID: 40678539

[ref26] Garcia-Lopez L. J. Jiménez-Vázquez D. Muela-Martinez J. A. Piqueras J. A. Espinosa-Fernandez L. Canals-Sans J.. (2024). Effectiveness of a transdiagnostic indicated preventive intervention for adolescents at high risk for anxiety and depressive disorders. Curr. Psychol. 43, 15484–15498. doi: 10.1007/s12144-023-05421-3

[ref27] Goodman R. (1997). The strengths and difficulties questionnaire: a research note. Child Psychol. Psychiatry 38, 581–586. doi: 10.1111/j.1469-7610.1997.tb01545.x, PMID: 9255702

[ref28] Gratz K. L. Roemer L. (2024). Multidimensional assessment of emotion regulation and dysregulation: development, factor structure, and initial validation of the difficulties in emotion regulation scale. J. Psychopathol. Behav. Assess. 26, 41–54. doi: 10.1023/B:JOBA.0000007455.08539.94

[ref29] Heeren A. Asmundson G. J. G. (2023). Understanding climate anxiety: what decision-makers, health care providers, and the mental health community need to know to promote adaptative coping. J. Anxiety Disord. 93:102654. doi: 10.1016/j.janxdis.2022.102654, PMID: 36414530

[ref30] Heeren A. Mouguiama-Daouda C. Contreras A. (2022). On climate anxiety and the threat it may pose to daily life functioning and adaptation: a study among European and African French-speaking participants. Clim. Chang. 173:15. doi: 10.1007/s10584-022-03402-2, PMID: 35912274 PMC9326410

[ref31] Hervás G. Jódar R. (2008). Adaptación al castellano de la Escala de dificultades en la regulación emocional. Clín. Sal. 19, 139–156.

[ref32] Hickman C. Marks E. Pihkala P. Clayton S. Lewandowski R. E. Mayall E. E.. (2021). Climate anxiety in children and young people and their beliefs about government responses to climate change: a global survey. Lancet Planet. Health 5, e863–e873. doi: 10.1016/s2542-5196(21)00278-3, PMID: 34895496

[ref33] Hogg T. L. Stanley S. K. O’Brien L. V. (2023). Synthesising psychometric evidence for the climate anxiety scale and Hogg eco-anxiety scale. J. Environ. Psychol. 88:102003. doi: 10.1016/j.jenvp.2023.102003

[ref34] Hogg T. L. Stanley S. K. O’Brien L. V. (2024). Validation of the hogg climate anxiety scale. Clim. Chang. 177:86. doi: 10.1007/s10584-024-03726-1

[ref9002] Hu L.-t. Bentler P. M. (1999). Cutoff criteria for fit indexes in covariance structure analysis: Conventional criteria versus new alternatives. Struct. Equ. Model. 6, 1–55. doi: 10.1080/10705519909540118

[ref35] Hwong A. R. Wang M. Khan H. Chagwedera D. N. Grzenda A. Doty B.. (2022). Climate change and mental health research methods, gaps, and priorities: a scoping review. Lancet Planet. Health 6, e281–e291. doi: 10.1016/s2542-5196(22)00012-2, PMID: 35278392

[ref36] Edizioni Centro Studi Erickson. (2022). Ecoansia: I cambiamenti climatici tra attivismo e paura. Trento, Italia: Edizioni Centro Studi Erickson.

[ref37] Innocenti M. Santarelli G. Faggi V. Castellini G. Manelli I. Magrini G.. (2021). Psychometric properties of the Italian version of the climate change anxiety scale. J. Clim. Change Health 3:100080. doi: 10.1016/j.joclim.2021.100080

[ref9003] Instituto Nacional de Estadística (INE). (2024). Censo Anual de Población. https://www.ine.es/dyngs/Prensa/CENSO2024.htm (Accessed January 15, 2025).

[ref38] Jang S. J. Chung S. J. Lee H. (2023). Validation of the climate change anxiety scale for Korean adults. Perspect. Psychiatr. Care 2023, 1–8. doi: 10.1155/2023/9718834

[ref39] Jaro’ah S. Saffana K. (2023). Adaptation of the climate anxiety scale in Indonesian version: the sample of young adults. Psikohumaniora J. Penelit. Psikol. 8, 309–328. doi: 10.21580/pjpp.v8i2.17462

[ref40] JASP Team. (2023). JASP (Version 0.18.1) [Computer software]. Available online at: https://jasp-stats.org/ (Accessed November 15, 2024).

[ref41] Jimenez-Vazquez D. Garcia-Lopez L. J. Piqueras J. A. Muela-Martinez J. A. Espinosa-Fernandez L. Vivas-Fernandez M.. (2024). Analyses of prediction, moderation, and mediation of a transdiagnostic, indicated preventive intervention (PROCARE-I) for adolescents at high risk of emotional problems. Evid. Based Pract. Child Adolesc. Ment. Health. 9, 1–17. doi: 10.1080/23794925.2024.238408838799772

[ref43] Kevorkian K. A. (2004). Environmental grief: hope and healing. Cincinnati, OH: Union Institute and University.

[ref44] Kurth C. Pihkala P. (2022). Eco-anxiety: what it is and why it matters. Front. Psychol. 13:981814. doi: 10.3389/fpsyg.2022.981814, PMID: 36211934 PMC9537110

[ref45] Larionow P. Sołtys M. Izdebski P. Mudło-Głagolska K. Golonka J. Demski M.. (2022). Climate change anxiety assessment: the psychometric properties of the polish version of the climate anxiety scale. Front. Psychol. 13:392. doi: 10.3389/fpsyg.2022.870392, PMID: 35645848 PMC9130850

[ref46] López-Cabanas M. Aragonés I. J. España J. (2019). Psicología y medioambiente: un reto ineludible. Pap. Psicol. 40:2908. doi: 10.23923/pap.psicol2019.2908

[ref48] McNally R. J. (2021). Network analysis of psychopathology: controversies and challenges. Annu. Rev. Clin. Psychol. 17, 31–53. doi: 10.1146/annurev-clinpsy-081219-092850, PMID: 33228401

[ref9004] Mead E. Roser-Renouf C. Rimal R. N. Flora J. A. Maibach E. W. Leiserowitz A. (2012). Information seeking about global climate change among adolescents: The role of risk perceptions, efficacy beliefs and parental influences. Atl. J. Commun. 20, 31–52. doi: 10.1080/15456870.2012.637027 22866024 PMC3411115

[ref49] Milfont T. L. Zubielevitch E. Milojev P. Sibley C. G. (2021). Ten-year panel data confirm generation gap but climate beliefs increase at similar rates across ages. Nat. Commun. 12:4038. doi: 10.1038/s41467-021-24245-y, PMID: 34230472 PMC8260718

[ref50] Mouguiama-Daouda C. Blanchard M. A. Coussement C. Heeren A. (2022). On the measurement of climate change anxiety: French validation of the climate anxiety scale. Psychol. Belg. 62, 123–135. doi: 10.5334/pb.1137, PMID: 35414943 PMC8954884

[ref51] Nori-Sarma A. Galea S. (2024). Climate change and mental health: a call for a global research agenda. Lancet Psychiatry 11, 316–317. doi: 10.1016/S2215-0366(24)00098-1, PMID: 38631783

[ref52] Notario-Pacheco B. Solera-Martínez M. Serrano-Parra M. D. Bartolomé-Gutiérrez R. García-Campayo J. Martínez-Vizcaíno V. (2011). Reliability and validity of the Spanish version of the 10-item Connor-Davidson resilience scale (10-item CD-RISC) in young adults. Health Qual. Life Outcomes 9:63. doi: 10.1186/1477-7525-9-63, PMID: 21819555 PMC3173284

[ref53] Ogunbode C. A. Doran R. Hanss D. Ojala M. Salmela-Aro K. Van Den Broek K. L.. (2022). Climate anxiety, wellbeing and pro-environmental action: correlates of negative emotional responses to climate change in 32 countries. J. Environ. Psychol. 84:101887. doi: 10.1016/j.jenvp.2022.101887

[ref54] Ojala M. (2012). Regulating worry, promoting hope: how do children, adolescents, and young adults cope with climate change? Int. J. Environ. Sci. Educ. 7, 537–561.

[ref55] Ojala M. (2022). How do children, adolescents, and young adults relate to climate change? Implications for developmental psychology. Eur. J. Dev. Psychol. 20, 929–943. doi: 10.1080/17405629.2022.2108396

[ref56] Orrù L. Taccini F. Mannarini S. (2024). Worry about the future in the climate change emergency: a mediation analysis of the role of eco-anxiety and emotion regulation. Behav. Sci. 14:255. doi: 10.3390/bs14030255, PMID: 38540558 PMC10967985

[ref57] Ortuño-Sierra J. Fonseca-Pedrero E. Paíno M. Aritio-Solana R. (2014). Prevalence of emotional and behavioral symptomatology in Spanish adolescents. Rev. Psiquiatr. Salud Ment. 7, 121–130. doi: 10.1016/j.rpsm.2013.12.003, PMID: 24530346

[ref58] Patrick R. Snell T. Gunasiri H. Garad R. Meadows G. Enticott J. (2022). Prevalence and determinants of mental health related to climate change in Australia. Aust. N. Z. J. Psychiatry 57, 710–724. doi: 10.1177/00048674221107872, PMID: 35785997

[ref59] Piqueras J. A. Pineda D. Martin-Vivar M. Sandín B. (2017). Confirmatory factor analysis and psychometric properties of the revised child anxiety and depression scale−30 (RCADS-30) in clinical and non-clinical samples. Rev. Psicopatol. Psicol. Clin. 22, 183–196. doi: 10.5944/rppc.vol.22.num.3.2017.19332

[ref60] Plohl N. Mlakar I. Musil B. Smrke U. (2023). Measuring young individuals’ responses to climate change: validation of the Slovenian versions of the climate anxiety scale and the climate change worry scale. Front. Psychol. 14:1297782. doi: 10.3389/fpsyg.2023.1297782, PMID: 38106391 PMC10722263

[ref61] Proulx K. Daelmans B. Baltag V. Banati P. (2024). Climate change impacts on child and adolescent health and well-being: a narrative review. J. Glob. Health 14:04061. doi: 10.7189/jogh.14.04061, PMID: 38781568 PMC11115477

[ref62] Ramadan R. Randell A. Lavoie S. Gao C. X. Manrique P. C. Anderson R.. (2023). Empirical evidence for climate concerns, negative emotions and climate-related mental ill-health in young people: a scoping review. Early Interv. Psychiatry 17, 537–563. doi: 10.1111/eip.13374, PMID: 36641809

[ref63] Ravens-Sieberer U. Erhart M. Rajmil L. Herdman M. Auquier P. Bruil J.. (2010). Reliability, construct and criterion validity of the KIDSCREEN-10 score: a short measure for children and adolescents' well-being and health-related quality of life. Qual. Life Res. 19, 1487–1500. doi: 10.1007/s11136-010-9706-5, PMID: 20668950 PMC2977059

[ref9005] Ravens-Sieberer U. Gosch A. Abel T. Auquier P. Bellach B. M. Bruil J.. (2001). Quality of life in children and adolescents: A European public health perspective. Soz Präventivmed. 46, 294–302. doi: 10.1007/BF01321080 11759336

[ref64] Sandín B. Chorot P. Valiente R. M. Chorpita B. F. (2010). Development of a 30-item version of the revised child anxiety and depression scale. Rev. Psicopatol. Psicol. 15, 165–178. doi: 10.5944/rppc.vol.15.num.3.2010.4095

[ref66] Sciberras E. Fernando J. W. (2021). Climate change-related worry among Australian adolescents: an eight-year longitudinal study. Child Adolesc. Ment. Health. 27, 22–29. doi: 10.1111/camh.12521, PMID: 34766705

[ref67] Searle K. Gow K. (2010). Do concerns about climate change lead to distress? Int. J. Clim. Chang. Strateg. Manag. 2, 362–379. doi: 10.1108/17568691011089891

[ref68] Simon P. D. Pakingan K. A. Aruta J. J. B. R. (2022). Measurement of climate change anxiety and its mediating effect between experience of climate change and mitigation actions of Filipino youth. Educ. Dev. Psychol. 39, 17–27. doi: 10.1080/20590776.2022.2037390

[ref71] Tam K. Chan H. Clayton S. (2023). Climate change anxiety in China, India, Japan, and the United States. J. Environ. Psychol. 87:101991. doi: 10.1016/j.jenvp.2023.101991

[ref72] Teo S. M. Gao C. X. Brennan N. Fava N. Simmons M. B. Baker D.. (2023). Climate change concerns impact on young Australians’ psychological distress and outlook for the future. J. Environ. Psychol. 93:102209. doi: 10.1016/j.jenvp.2023.102209

[ref73] van de Schoot R. Lugtig P. Hox J. (2012). A checklist for testing measurement invariance. Eur. J. Dev. Psychol. 9, 486–492. doi: 10.1080/17405629.2012.686740

[ref74] Vandenberg R. J. Lance C. E. (2000). A review and synthesis of the measurement invariance literature: suggestions, practices, and recommendations for organizational research. Organ. Res. Methods 3, 4–70. doi: 10.1177/109442810031002

[ref75] Villalobos-Prats E. Neville T. Nadeau K. C. Campbell-Lendrum D. (2023). WHO academy education: globally oriented, multicultural approaches to climate change and health. Lancet Planet. Health 7, e10–e11. doi: 10.1016/s2542-5196(22)00252-2 36608940 PMC9834511

[ref76] Vivas-Fernandez M. Garcia-Lopez L.-J. Muela-Martinez J. A. Piqueras J. A. Espinosa-Fernandez L. Jimenez-Vazquez D.. (2024). Exploring the role of resilience as a mediator in selective preventive transdiagnostic intervention (PROCARE+) for adolescents at risk of emotional disorders. Eur. J. Psychol. Open 83, 21–34. doi: 10.1024/2673-8627/a000054

[ref77] Vivas-Fernandez M. Garcia-Lopez L. J. Piqueras J. A. Espinosa-Fernandez L. Muela-Martinez J.-A. Jimenez-Vazquez D.. (2023b). A 12-month follow-up of PROCARE+, a transdiagnostic, selective, preventive intervention for adolescents at-risk for emotional disorders. Child Psychiatry Hum. Dev. doi: 10.1007/s10578-023-01638-2 PMC12504353 38127203

[ref78] Vivas-Fernandez M. Garcia-Lopez L. J. Piqueras J. A. Muela-Martinez J. A. Canals-Sans J. Espinosa-Fernandez L.. (2023a). Randomized controlled trial for selective preventive transdiagnostic intervention for adolescents at risk for emotional disorders. Child Adolesc. Psychiatry Ment. Health 17:77. doi: 10.1186/s13034-023-00616-9, PMID: 37353831 PMC10290361

[ref80] Wullenkord M. C. Tröger J. Hamann K. R. S. Loy L. S. Reese G. (2021). Anxiety and climate change: a validation of the climate anxiety scale in a German-speaking quota sample and an investigation of psychological correlates. Clim. Chang. 168, 1–23. doi: 10.1007/s10584-021-03234-6

[ref9006] Zelezny L. C. Chua P.-P. Aldrich C. (2000). New ways of thinking about environmentalism: Elaborating on gender differences in environmentalism. J. Soc. Issues. 56, 443–457. doi: 10.1111/0022-4537.00177

