# Supplementary material for: Psychometric properties of the Spanish version of the climate anxiety scale in Spanish-speaking adolescents
Source: Front Psychol. 2025 Aug 5;16:1631481. doi: 10.3389/fpsyg.2025.1631481 (PMC12361156; doi:10.3389/fpsyg.2025.1631481)

Supplementary Table 1.

Centrality study variables relationship network: Emotional variables and Overall CAS score

	Betweenness	Closeness	Strength
Overall CAS score	-0.85	−2.153	−1.916
RCADS_MD	-0.56	0.340	0.819
RCADS_PD	-0.846	0.122	0.668
RCADS_SoP	0.00	0.582	0.281
RCADS_SAD	0.56	−0.395	−1.172
RCADS_GAD	1.97	0.980	0.176
RCADS_OCD	0.56	0.827	0.766
DERS	-0.85	−0.304	0.378

Note. RCADS-MD: Major Depressive Disorder subscale of the RCADS; RCADS-PD: Panic Disorder subscale of the RCADS; RCADS-SoP: Social Phobia subscale of the RCADS; RCADS-SAD: Separation Anxiety Disorder subscale of the RCADS; RCADS-GAD: Generalized Anxiety Disorder subscale of the RCADS; RCADS-OCD: Obsessive-Compulsive Disorder subscale of the RCADS; DERS: Difficulties in Emotion Regulation Scale

Supplementary Table 2.

Centrality study variables relationship network: Emotional variables and *CAS subscales: Cognitive-emotional impairment and* Functional impairment.

	Betweenness	Closeness	Strength
Cognitive-emotional impairment subscale	0.17	−1.68	−0.43
Functional impairment subscale	−0.21	−1.72	−0.62
RCADS_MD	−0.95	0.59	1.03
RCADS_PD	−1.32	0.23	0.73
RCADS_SoP	−0.58	0.44	0.12
RCADS_SAD	0.91	0.05	−2.19
RCADS_GAD	2.02	1.01	0.01
RCADS_OCD	−0.21	0.63	0.88
DERS	0.17	0.46	0.48

Note. CAS_TOTAL: Overall CAS score; RCADS-MD: Major Depressive Disorder subscale of the RCADS; RCADS-PD: Panic Disorder subscale of the RCADS; RCADS-SoP: Social Phobia subscale of the RCADS; RCADS-SAD: Separation Anxiety Disorder subscale of the RCADS; RCADS-GAD: Generalized Anxiety Disorder subscale of the RCADS; RCADS-OCD: Obsessive-Compulsive Disorder subscale of the RCADS; DERS: Difficulties in Emotion Regulation Scale

Supplementary Figure 1. Centrality Plots for network depicting the betweenness, closeness, and degree (strength), expected influence of each node (variable: Emotional variables and Overall CAS score).

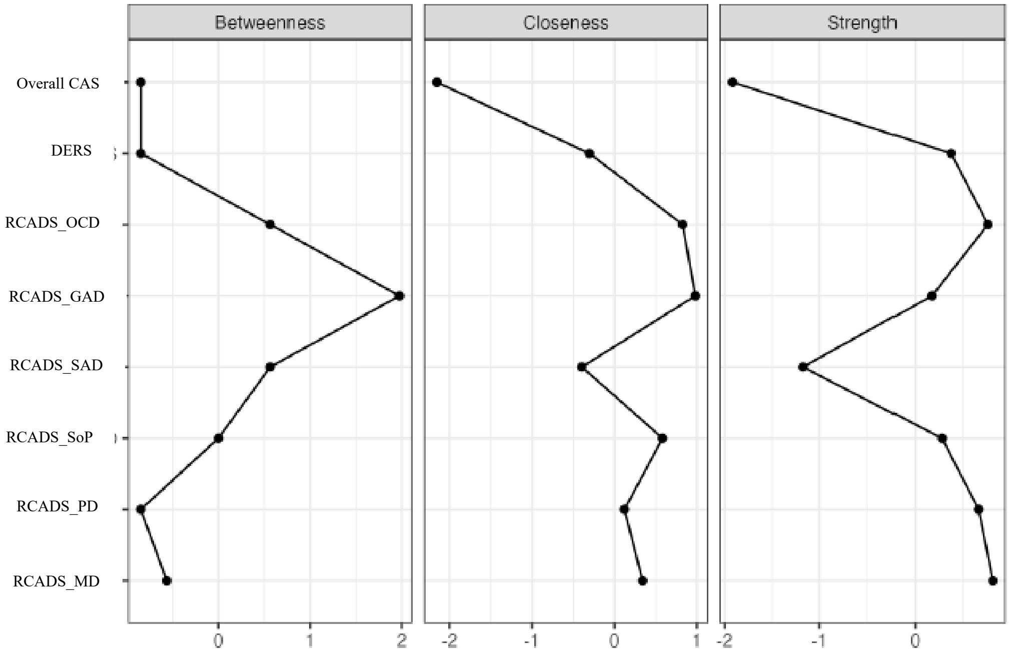

Supplementary Figure 2. Centrality Plots for network depicting the betweenness, closeness, and degree (strength), expected influence of each node (variable: Emotional variables and *CAS subscales: Cognitive-emotional impairment and* Functional impairment).

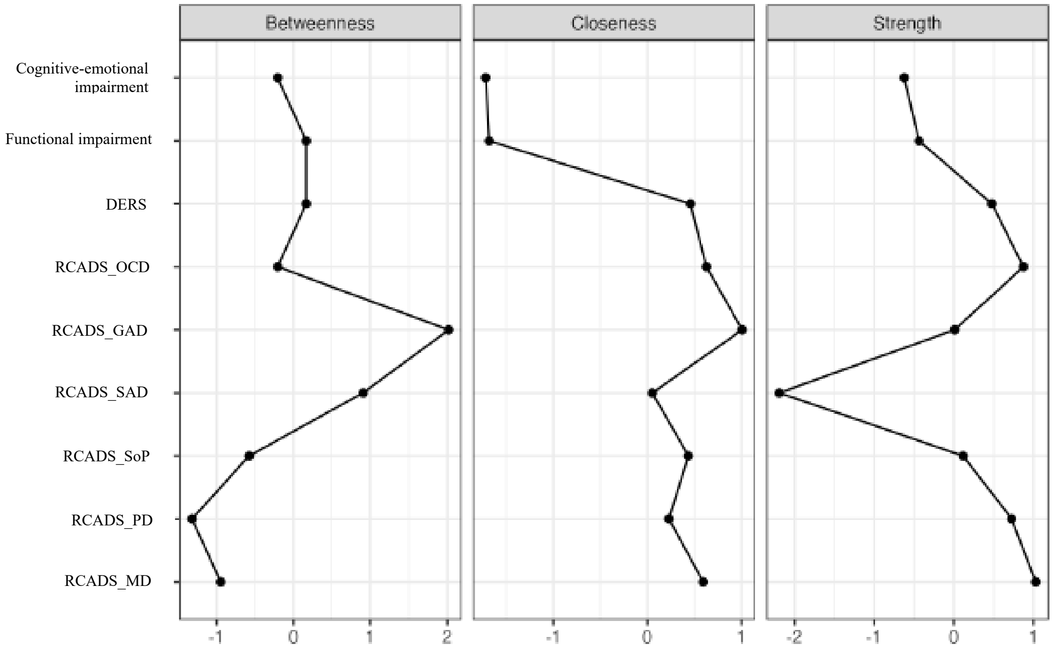

Supplement: Supplementary file 1 [file Data_Sheet_1.pdf]
